# 

**Published:** 1916-04

**Authors:** 


### Publishers' Special Announcements

Blowing one's horn is one thing and publication of a fact is another. One is not a synonym for the other. Advertising today is not a blowing of horns, it is a statement of fact. The goods delivered prove the statement and make it a fact.

Users of goods are interested to learn about them and to know where to get them. It is up to the manufacturers to make a statement of fact regarding their goods in order that the purchaser may know about them. Advertising is the medium through which buyer and seller get together. One wants to buy and the other desires to sell. It is apparent that a statement that misrepresents an article of merchandise could not stand the test of advertisement year after year.

The well known firms that advertise in this journal are not blowing horns but are simply making their monthly statement of facts.

### ANNOUNCEMENTS

NORTHERN OHIO DENTAL ASSOCIATION. Meets in Cleveland, June 1 to 3, 1916.

Sandusky, Ohio. C. D. PECK, *Secy.*

ILLINOIS STATE MEETING. Will be held May 9 to 12, 1916, in Springfield, Ill.

Quincy, Ill. H. L. WHIPPLE, *Secy.*

INDIANA STATE SOCIETY MEETING. Will be held in Indianapolis, May 16 to 18, 1916, in Claypool Hotel.

Lafayette, Ind. A. R. ROSS, *Secy.*

CONNECTICUT STATE DENTAL ASSOCIATION. Meets June 13 to 15, 1916, in New London, at the Griswold Hotel. This is a beautiful place for the meeting and a good pragram and fine time is promised for every one.

New Haven, Conn. E. R. BRYANT, *Secy.*

TEACHER'S INSTITUTE OFFICERS. At the meeting in Minneapolis the following officers were chosen for the year: Dr. S. W. Bowles, Washington, D. C., President; Dr. J. F. Biddle, Pittsburgh, Pa., Vice President; Dr. Abram Hoffman, 529 Franklin St., Buffalo, N. Y., Secretary-Treasurer. Drs. A. W. Thorton, R. W. Bunting and A. D. Black, Executive Board. The next meeting will be held in Philadelphia, January 23 to 25, 1917.

MICHIGAN STATE BOARD OF DENTAL EXAMINERS. The next regular meeting of the Michigan State Board of Dental Examiners for the examination of applicants who may desire to practice dentistry in Michigan, will be held in the Dental College at Ann Arbor, beginning Monday, June 19, 1916, at 8 o'clock, a. m., and will continue through Saturday, June 24th. For application blanks and all further information application should be made to,

Stephenson, Mich. E. O. GILLESPIE, *Secy-Treas.*

